# Gene flow and genetic structure of *Bactrocera carambolae* (Diptera, Tephritidae) among geographical differences and sister species, *B. dorsalis*, inferred from microsatellite DNA data

**DOI:** 10.3897/zookeys.540.10058

**Published:** 2015-11-26

**Authors:** Nidchaya Aketarawong, Siriwan Isasawin, Punchapat Sojikul, Sujinda Thanaphum

**Affiliations:** 1 Department of Biotechnology, Faculty of Science, Mahidol University, 272 Rama VI Road, Phayathai, Bangkok, 10400 THAILAND; 2 equal contribution

**Keywords:** Carambola fruit fly, species complex, gene flow, incipient species, pest control, SIT, Salaya5 strain

## Abstract

The Carambola fruit fly, *Bactrocera carambolae*, is an invasive pest in Southeast Asia. It has been introduced into areas in South America such as Suriname and Brazil. *Bactrocera carambolae* belongs to the *Bactrocera dorsalis* species complex, and seems to be separated from *Bactrocera dorsalis* based on morphological and multilocus phylogenetic studies. Even though the Carambola fruit fly is an important quarantine species and has an impact on international trade, knowledge of the molecular ecology of *Bactrocera carambolae*, concerning species status and pest management aspects, is lacking. Seven populations sampled from the known geographical areas of *Bactrocera carambolae* including Southeast Asia (i.e., Indonesia, Malaysia, Thailand) and South America (i.e., Suriname), were genotyped using eight microsatellite DNA markers. Genetic variation, genetic structure, and genetic network among populations illustrated that the Suriname samples were genetically differentiated from Southeast Asian populations. The genetic network revealed that samples from West Sumatra (Pekanbaru, PK) and Java (Jakarta, JK) were presumably the source populations of *Bactrocera carambolae* in Suriname, which was congruent with human migration records between the two continents. Additionally, three populations of *Bactrocera dorsalis* were included to better understand the species boundary. The genetic structure between the two species was significantly separated and approximately 11% of total individuals were detected as admixed (0.100 ≤ *Q* ≤ 0.900). The genetic network showed connections between *Bactrocera carambolae* and *Bactrocera dorsalis* groups throughout Depok (DP), JK, and Nakhon Sri Thammarat (NT) populations. These data supported the hypothesis that the reproductive isolation between the two species may be leaky. Although the morphology and monophyly of nuclear and mitochondrial DNA sequences in previous studies showed discrete entities, the hypothesis of semipermeable boundaries may not be rejected. Alleles at microsatellite loci could be introgressed rather than other nuclear and mitochondrial DNA. *Bactrocera carambolae* may be an incipient rather than a distinct species of *Bactrocera dorsalis*. Regarding the pest management aspect, the genetic sexing Salaya5 strain (SY5) was included for comparison with wild populations. The SY5 strain was genetically assigned to the *Bactrocera carambolae* cluster. Likewise, the genetic network showed that the strain shared greatest genetic similarity to JK, suggesting that SY5 did not divert away from its original genetic makeup. Under laboratory conditions, at least 12 generations apart, selection did not strongly affect genetic compatibility between the strain and wild populations. This knowledge further confirms the potential utilization of the Salaya5 strain in regional programs of area-wide integrated pest management using SIT.

## Introduction

*Bactrocera carambolae* Drew & Hancock, the Carambola fruit fly, is a key insect pest belonging to the *Bactrocera dorsalis* species complex (Diptera, Tephritidae). Its native distribution covers the western part of the Indo-Australian Archipelago (determined by Wallace’s and Huxley’s lines), including the Thai/Malay Peninsula and Western Indonesia (White and Elson-Harris 1992, Clarke et al. 2005, EPPO 2014). Outside of Southeast Asia, the Carambola fruit fly was originally misidentified as *Dacus dorsalis* Hendel (Drew 1989), but later recognized as a separate species and named *Bactrocera carambolae*. The fly was firstly recorded to be a trans-continental pest in Paramaribo, Suriname, in 1975. Supposedly, tourists and trade by air between Indonesia and Suriname introduced the pest during the 1960s and 1970s. *Bactrocera carambolae* was also reported in other areas of South America: in 1986 in French Guyana (approximately 200 km from Paramaribo); in 1993 in Orealla, Guyana, at the border of Suriname (approximately 220 km from Paramaribo); and in 1996 in the Brazilian city of Oiapoque at the border with French Guyana (about 500 km from Paramaribo) (Malavasi et al. 2000, 2013). It can potentially spread to other countries in South America, Central America, and the Caribbean if effective control action is deficient (van Sauers-Mullers and Vokaty 1996). Currently, the Carambola fruit fly is in the process of being eradicated from the region of North Brazil (Malavasi et al. 2000, 2013). *Bactrocera carambolae* is regarded to be a polyphagous pest. It has a broad host range, including wild and cultivated fruits such as star fruit, mango, guava, and grapefruit (van Sauers-Mullers and Vokaty 1996, EPPO 2014). However, host plants for the fly were occasionally observed to be different between native and introduced areas as reported by van Sauers-Muller (2005). Despite being an important pest, knowledge of molecular ecology concerning species status and pest management is needed. There are no previous studies, except multilocus phylogeny (Boykin et al. 2014), comparing it to closely related species such as *Bactrocera dorsalis* (Hendel) (Aketarawong et al. 2007, 2011, 2014a, 2014b, Khamis et al. 2009, Wan et al. 2011, Shi et al. 2012, Schutze et al. 2012, Krosch et al. 2013).

Within the *Bactrocera dorsalis* species complex, *Bactrocera carambolae* is still valid, even though a few members (i.e., *Bactrocera papayae* Drew & Hancock, *Bactrocera philippinensis* Drew & Hancock, and *Bactrocera invadens* Drew, Tsuruta & White) of the complex were recently synonymized with *Bactrocera dorsalis* (Schutze et al. 2012, 2013, and review in Schuzte et al. 2015). Based on a few species concepts, *Bactrocera carambolae* is distinct from *Bactrocera dorsalis*. For example, differences of morphology and morphometric data (Drew and Hancock 1994, Iwahashi 1999, Drew and Romig 2013) as well as monophyly confirmed by sequencing analyses of nuclear and mitochondrial DNA (Armstrong and Cameron 2000, Armstrong and Ball 2005, Boykin et al. 2014) were evidence to support morphological and phylogenetic species concepts, respectively. With regard to the biological species concept, pre- and post-reproductive isolation between the two species were also reported. Variations of reproductive morphologies (Iwahashi et al. 1999), host plants (Drew et al. 2008), and male pheromone components (Wee and Tan 2005b) and consumption doses (Wee and Tan 2005a) may reduce the number of sexual encounters between the two species. Furthermore, differences of mating times (McInnis et al. 1999, Schutze et al. 2013) and behavior (Schutze et al. 2013) account for reducing mating success. Nevertheless, inadequate reproductive isolation through hybridization was sometimes observed; viable *F*1 and further generations were produced under semi-natural conditions (Isasawin et al. 2014). Intermixing of pheromone components was consistently observed in semi-natural (Isasawin et al. 2014) and natural conditions (Wee and Tan 2005b). Slightly different genomes between the two species were observed in samples from inbreeding experiments using cytogenetic analysis (Augustinos et al. 2014). As such, the study of species status of *Bactrocera carambolae* and *Bactrocera dorsalis* is still an interesting issue. This information has implications not only for research but also for pest management and quarantine policies (Schutze et al. 2015).

In order to manage fruit fly pests, a method such as the Sterile Insect Technique (SIT) is commonly used to prevent, suppress, eradicate, or contain these pests (Klassen and Curtis 2005). In principle, male individuals are mass-sterilized and released into the target area. They competitively seek and mate with target fertile females. This leads to the production of nonviable offspring and subsequently suppresses the population. SIT is thus considered to be a target-specific, environmentally clean, and suitable birth control method. However, to enhance the effectiveness of SIT programs, the desired insect strains for irradiation and release are male-only strains (known as Genetic Sexing Strains or GSSs). For GSSs, male individuals can be sex-sorted before irradiation and release steps. An available GSS for *Bactrocera carambolae* has been successfully developed and evaluated, named Salaya5 (Isasawin et al. 2014). This strain was proven to competitively mate with two wild populations from Jakarta and North Sumatra, Indonesia, in field cage conditions. Nonetheless, for long-term pest control, a genetic compatibility between the mass-rearing colony (mother colony of released sterile insects) and wild population needs to be routinely monitored (Krafsur 2005, Cerritos et al. 2012). The genetic compatibility may drive mate choice and fertilization, especially in polyandrous pests wherein females have a post-mating opportunity to choose sperm from several males (review in Tregenza and Wedell 2000). Many invasive fruit fly pests are polyandrous such as *Bactrocera dorsalis*, *Bactrocera tryoni* (Froggatt), or *Ceratitis capitata* (Wiedemann) (Shelly and Edu 2008). Mating incompatibility between wild and released populations could result in an ineffective SIT program such as was the case for the New World screwworm in Jamaica (McDonagh et al. 2009).

Microsatellite DNA markers are a useful tool for population genetic and molecular ecological studies as well as pest management. The sequences of microsatellites are short tandem repeats that are widely distributed throughout the entire eukaryotic genome. Microsatellite loci selected for population genetics are Mendelian inherited, neutral, and polymorphic. Such markers generally provide a more contemporary estimate of diversity/structure because they mutate quicker and present a co-dominant feature, unlike mitochondrial DNA or other nuclear DNA markers (Hoshino et al. 2012). Likewise, using the genetic cluster approach based on microsatellite data can resolve intra- and interspecific relationships. Several microsatellite markers for invasive tephritid fruit flies were therefore established for studying population genetics in different geographical regions to infer colonization process (e.g., *Ceratitis capitata* (Bonizzoni et al. 2001, 2004, Meixer et al. 2002), *Bactrocera dorsalis* (Aketarawong et al. 2007, 2014a, Khamis et al. 2009, Wan et al. 2011, Shi et al. 2012), *Zeugodacus cucurbitae* (Coquillett) (Virgilio et al. 2010, Wu et al. 2011), *Bactrocera oleae* (Gmelin) (Nardi et al. 2005, Zygoridis et al. 2009, Dogaç et al. 2013)). In addition, established markers were used for solving species status in members of species complexes. For example, *Bactrocera dorsalis* and its synonym *Bactrocera papayae* were identified to have a single genetic cluster (Krosch et al. 2013) or weak population’s genetic structure with no specific alleles (Aketarawong et al. 2014b), suggesting a single entity. However, members of the *Ceratitis* FAR complex were genetically divided, belonging to their species’ genetic clusters, and some individuals were identified to be hybrid individuals, supporting a lack of reproductive isolation (Virgilio et al. 2013). Although isolated and developed for one species, the microsatellite primer sets can sometimes be used on related species (review in Barbara et al. 2007, Hoshino et al. 2012). Because of the conservation of microsatellite DNA sequences’ flanking region across related species, cross-amplification is possibly an alternative approach for species whose data regarding microsatellite markers are unavailable.

The aim of this research, therefore, is to study the population genetics of *Bactrocera carambolae*, using modified cross-species amplification of microsatellite DNA markers derived from *Bactrocera dorsalis* and the junior synonym, *Bactrocera papayae*, with regard to three aspects of species status and pest management. Intra-specific variation was analyzed among seven populations, consisting of native and trans-continentally introduced populations, for inference of colonization processes. Moreover, samples of *Bactrocera dorsalis* were included to examine the population genetic structure and as an attempt to better understand the species boundary between *Bactrocera carambolae* and *Bactrocera dorsalis*. Lastly, concerning pest management aspect, we validated the potential for use the genetic sexing Salaya5 strain in regional SIT programs. The Salaya5 were genotyped and genetically compared to other wild *Bactrocera carambolae* populations, in order to evaluate genetic compatibility between them.

## Methods

### Sample collections

**Figure 1.**
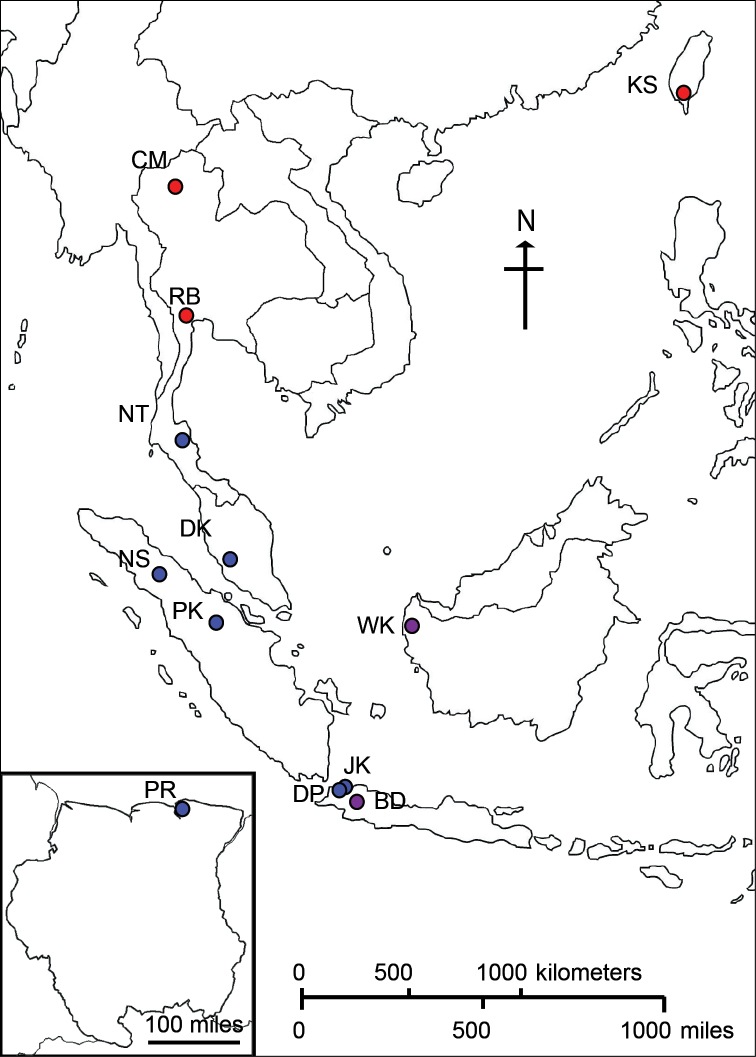
Sampling collections of *Bactrocera carambolae* and *Bactrocera dorsalis* in this study. Seven populations of *Bactrocera carambolae* (blue dots) were collected from Southeast Asia and Suriname. Three populations of *Bactrocera dorsalis* (red dots) were sampled from East and Southeast Asia. Two other unidentified populations (purple dots) were included. Information for each population is described in Table 1.

Nine wild fruit fly populations were collected from four geographical areas: Indonesia (6), Malaysia (1), Thailand (1), and Suriname (1) (Table 1 and Figure 1). These populations were from hosts and locations within the known ranges of *Bactrocera carambolae* (http://www.cabi.org/isc/datasheet/8700). In Indonesia, six populations were collected from three main islands; two populations (i.e., North Sumatra-NS and Pekanbaru-PK) were sampled from Sumatra; three populations (i.e., Depok-DP, Jakarta-JK, and Bandung-BD) were sampled from Java; and another (West Kalimantan-WK) was sampled from Borneo. In Thailand, one population was collected from the southern region (Nakhon Sri Thammarat-NT). All fruit fly samples were firstly characterized as *Bactrocera carambolae* based on Drew and Hancock (1994). In addition, the male pheromone profile (Wee and Tan 2005b) and/or ITS1 marker (Armstrong and Cameron 2000, Boykin et al. 2014) were also used to confirm the characterization (Table 1). Only populations BD and WK showed conflicting identifications and were classified as unidentified populations.

**Table 1.** Sample collection used in this study

Sample name	Type	Population characterization							
		Morphology^1^		Location^2^	Host plant ^3^	Male pheromone^4^		ITS1^5^	
		*Bcar*	*Bdor*			*Bcar*	*Bdor*	*Bcar*	*Bdor*
NS	Alive	x		North Sumatra, Indonesia (01°47'N; 099°02'E)	*Averrhoa carambola*	x		x	
PK	Dead	x		Pekanbaru, Riau, Indonesia (00°32'N; 101°27'E)	n/a			x	
DP	Dead	x		Depok, West Java, Indonesia (06°23'S; 106°49'E)	*Averrhoa carambola*			x	
JK	Alive	x		Jakarta, Indonesia (06°14'S; 106°49'E)	*Averrhoa carambola*	x		x	
BD	Dead	x		Bandung, West Java, Indonesia (06°54'S; 107°36'E)	n/a				x
WK	Dead	x		West Kalimantan, Indonesia (00°02'S; 109°19'E)	n/a				x
DK	Dead	x		Dengkil, Selangor, Malaysia (03°20'N; 101°30'E)	n/a			x	
NT	Alive	x		Nakhon Sri Thammarat, Thailand (08°19'N; 099°57'E)	n/a	x		x	
PR	Dead	x		Paramaribo, Suriname (05°52'N; 055°10'W)	n/a			x	
RB	Alive		x	Ratchaburi, Thailand (13°52'N; 099°48'E)	*Mangifera indica*		x		x
CM	Dead		x	Chang Mai, Thailand (18°47'N; 098°59'E)	n/a				x
KS	Dead		x	Kaohsiung, Taiwan (23°02'N; 120°35'E)	*Mangifera indica*				x
SY5	Alive	x		Thailand (Lab) (Introgression strain)	-	x		x	

1 followed the method of Drew and Hancock (1994)

2 covered known distribution of *Bactrocera carambolae* and *Bactrocera dorsalis* (http://www.cabi.org/isc/)

3 observed in the collection area

4 followed the method of Wee and Tan (2005b)

5 followed the method of Armstrong and Cameron (2000) and Boykin et al. (2014)

n/a no available data

Three other populations of *Bactrocera dorsalis* were included in this study as outgroup samples for the investigation of genetic relationship between two cryptic species. These populations were collected from the known distributions of *Bactrocera dorsalis* (http://www.cabi.org/isc/datasheet/17685) and characterized as *Bactrocera dorsalis* using the same methods described before (Table 1). One population is from Ratchaburi, Thailand (RB), which is a representative source of *Bactrocera dorsalis* in Southeast Asia (Aketarawong et al. 2007, 2014a). Another is from the northern part of Thailand, Chang Mai (CM). The other was collected from Kaohsiung, Taiwan (KS). This sample is supposed to have been introduced from mainland China and has become an isolated population (Aketarawong et al. 2007, 2014a).

To record the genetic relationship between the genetic sexing Salaya5 strain (Isasawin et al. 2014) and wild populations, a sample of the Salaya5 colony (SY5) was included. The Salaya5 strain was created by hybridization and introgression of the genetic sexing *Bactrocera dorsalis* strain, named Salaya1 (Isasawin et al. 2012), and *Bactrocera carambolae* from Jakarta, Indonesia. This strain has a genetic background close to *Bactrocera carambolae* (99.9%) and a part of the Y-pseudo linked autosome carrying a dominant allele of white pupae alleles of Salaya1 strain (Isasawin et al. 2014). The strain was confirmed to be *Bactrocera carambolae* described by Isasawin et al. (2014). The Salaya5 colony has been maintained in the conditions presented in Isasawin et al. (2014).

All samples were preserved in 95% ethanol and kept at -20 °C until use. The genomic DNA of each fly was extracted using the method of Baruffi et al. (1995).

### Development of modified cross-species amplification of microsatellite DNA markers

Twelve microsatellite loci (Bd1, Bd9, Bd15, Bd19, Bd39, Bd42, and Bd85B derived from *Bactrocera dorsalis* s.s. (Aketarawong et al. 2006), and Bp58, Bp73, Bp125, Bp173, and Bp181 derived from *Bactrocera papayae* (Shearman et al. 2006)), previously established for study of members in the same complex (Aketarawong et al. 2014b), were analyzed in *Bactrocera carambolae*. Amplifications were set up in a 15- μl volume reaction containing 1 × buffer, 2.5 mM MgCl_2_, 25 μM dNTPs, 0.5 U *Taq* polymerase (Vivantis), 5 μM of each primer, and 100 ng of genomic DNA. PCRs were performed using the thermal cycler Flexcycler (AnalytikJena, Germany) using the conditions described by Aketarawong et al. (2014b). Amplicons of sizes consistent with fragments of *Bactrocera dorsalis* were cloned and sequenced using an ABI PRISM 310 genetic analyser (Macrogen, Korea). All sequences, except Bp173, showed homology to the original sequences in Genbank (Table 2).

**Table 2.** Microsatellite loci motif and primers used in this study.

**Locus**	**Repeat motif [GenBank Accession no.**]	**Original motif in *Bactrocera dorsalis*^1^ and *Bactrocera papayae*^2 ^[Genbank Accession no.**]	**Primer (5’-3’)**	***T*_a_ (°C)**	***n*_a_**	**Size range in bp** (*n***_a_)**		
						Bcar^*^	*Bdor*	SY5
Bcar1	CT(CA)_4_CGCA	CT(CA)_4_CG(CA)_2_	F: TGCTTAACAGTAATTGCTCCTT	62	11	96–112	96–108	100–108
	[KT355774]	[DQ482030]^1^	R: AAGCAGTAAACAATAAAGTTCCAA			(9)	(7)	(4)
Bcar9	(GT)_2_AA(GT)_6_GA	GA(GT)_7_GA	F: GCTGATATGTGTGCGTCTTATTTGTGA	69	16	156–182	140–172	168–186
	[KT355775]	[DQ482033]^1^	R: ATCTCGTATTGTGGTTGCTTAAATATG			(12)	(8)	(6)
Bcar15	(CA)_3_CC(CG)_2_CAA (CA)_6_CGTG(TACA)_3_	(CA)_8_CGCAA (CA)_4_CGTG(TACA)_3_	F: TGCCTTGTGCTATTTAATCTTTATCAA	63	12	183–199	155–195	191–195
	[KT355776]	[DQ482034]^1^	R: AAATAAACAAAACAAAATGCAAATACA			(9)	(7)	(2)
Bcar19	(CA)_2_CT(CA)_6_(TA)_2_CA	(CA)_2_CT(CA)_6_(TA)_2_(CATA)_2_	F: TAGATGGAGATGGGTGCGTGTACATG	71	13	149–171	155–175	167
	[KT355777]	[DQ482035]^1^	R: GCGTGTTCACAAGGACTAATCGAA			(11)	(9)	(1)
Bcar39	(GT)_8_	(GT)_8_	F: GGTCAAACAAATCACTCAGTAAC	63	14	68–92	78–104	84–90
	[KT355778]	[DQ482037]^1^	R: CCGTTATATCAGGCAAATCTATA			(8)	(11)	(4)
Bcar42	CAAA(CA)_2_AA(CA)_3_(TA)_4_	(CA)_7_(TA)_7_TG(TA)_2_GC(CA)_3_TA	F: GCACAGTGAGCGTTACAAG	64	13	150–190	172–186	180–186
	[KT355779]	[DQ482038]^1^	R: TGTTTTTACAGTTATACACTTCCCT			(10)	(8)	(4)
Bcar73	(GT)_9_	(GT)_5_	F: AGCGAAAACCAACTACTACCG	67	7	107–119	109–115	113–115
	[KT355780]	[AY847272]^2^	R: CCACTACTTCATCTTGTTCCTGCAG			(7)	(4)	(2)
Bcar181	(AC)_5_ATAC	(AC)_8_	F: GTGCATGCCTTCGTGTAGCCTAACTCA	67	5	101–109	103–109	103–105
	[KT355781]	[AY847280]^2^	R: AATCTGCGAAGGATATCAACCATTCAC			(5)	(4)	(2)

1 Aketarawong et al. (2006)

2 Shearman et al. (2006)

* These data are calculated using seven *Bactrocera carambolae* populations

To improve null allele problems due to mutations on primer-binding sites and/or unsuitable PCR conditions, a new set of primers were designed and renamed for the 11 loci using OLIGO version 4.0-s (Rychlik and Rhoads 1989). Moreover, the new annealing temperature for each primer pair is shown in Table 2.

Fifteen flies from Jakarta and North Sumatra, Indonesia were initially screened with the 11 sets of new primers using the PCR conditions mentioned above. Electrophoresis and allele scoring were determined as in Aketarawong et al. (2014b). Only eight loci were selected based on sharpness, specificity, and polymorphism of amplified products for further testing in all samples (Table 2).

### Genetic variations

The descriptive parameters of population genetics were estimated using GENALEX v.6.5 (Peakall and Smouse 2012). These parameters include the mean number of alleles (*n*_a_), mean effective number of alleles (*n*_e_), mean number and frequency of private alleles (*n*_p_ and *A*_p_, respectively), and mean observed and expected heterozygosity (*H*_O_ and *H*_E_, respectively). Null allele frequency (*A*_n_) was estimated following Brookfield (1996). Departure from Hardy-Weinberg equilibrium and the effect of linkage disequilibrium were tested using GENEPOP v.4 (Rosset 2008), with their critical levels set according to the sequential Bonferroni test (Rice 1989).

### Population structure

Genetic differentiation (*F*_ST_) among 13 populations was measured using MICROSATELLITE ANALYSER (MSA) (Dieringer and Schlötterrer 2003). In addition, genetically distinct groups (or clusters) were determined using the Bayesian approach implemented in STRUCTURE v.2.3.1 (Pritchard et al. 2000, Falush et al. 2003). The admixture model (the *F* model), assuming correlated allele frequencies, was run. The program computed the number of possible clusters (*K*) from one to 13, with the condition of the burn-in period being 100,000 steps, followed by 500,000 MCMC repetitions. For each *K* value, five iterations were performed. The other parameters were set at default values: a standard deviation of 0.05, prior *F*_ST_ mean of 0.01, and different values of *F*_ST_ for different subpopulations. The optimal number of hypothetical clusters was indicated by the Delta *K* method (Evanno et al. 2005). To identify the potential admixed individuals between *Bactrocera carambolae* and *Bactrocera dorsalis*, an individual-based genetic cluster was plotted. STRUCTURE analysis under different assumptions was also run to verify the consistency. The repeated analyses considering (1) uncorrelated allele frequency and (2) missing data as recessive homozygotes for the null alleles were set to avoid the shared descent of samples and to verify possible bias from null alleles, respectively.

Principle Coordinate Analysis (PCoA) was used to display genetic divergence among fruit fly populations in multidimensional space. This analysis was based on allele frequency data and performed on genetic distance using GENALEX v.6.5 (Peakall and Smouse 2012). The subprogram MOD3D in NTSYS-pc v.2.1 (Rohlf 2005) was subsequently used for plotting the first three principal coordinates.

To test genetic homogeneity in different hierarchical population structures, Analysis of Molecular Variance (AMOVA) in ARLEQUIN v.3.1.1 was used (Excoffier and Lischer 2010). After 1,000 permutations, populations were grouped according to nine criteria described in Table 5.

### Isolation by distance (IBD)

The correlation analysis between genetic and geographic distance was performed using the subprogram ISOLDE in the GENEPOP package (Rousset 2008).

### Genetic network analyses

Analyses of the genetic networks were performed using EDENetworks (Kivelä et al. 2015). This program is advantageous in that it can provide graphical representations of the structure and dynamics of a system of interaction between populations in multidimensional space, without *a priori* assumptions of the clustering of populations and some of the constraints (e.g., binary branching) compulsory in phylogenetic trees. Data types of ‘genotype matrix, diploid, sampling site based’ were used as input. The genetic distance metrics or network files were calculated using the *F*_ST_-based distance of Reynolds (Reynolds et al. 1983); networks were constructed.

Networks consist of nodes (or vertices), corresponding to populations, connected by links (or edges), representing their relationships or interactions. Connectivity degree (or Degree) is the number of edges connected to a node summarizing how strongly a population associated with the other populations in the system and whether or not it is a source population. Betweenness-centrality (*BC*) determines the relative importance of a node within the network as an intermediary in the flow of information. Each network was weighted demonstrating genetic similarity associated with each link.

The network can be analyzed at various meaningful thresholds (*thr*). *thr* is the maximum distance considered as generating a connection in the network. One meaningful distance is the one corresponding to the percolation threshold (*D*_p_), edges with weights below the threshold were removed from the weighted network, and only the most important links were retained. Above the *D*_p_ level, there is a giant component containing almost all the nodes in the networks while below the *D*_p _level, the network is fragmented into small disconnected components and the system therefore loses its ability to transport information across the whole system. Therefore, scanning at different thresholds was performed to analyze possible sub-structured systems to observe the sequential forms of clusters (Kivelä et al. 2015). The *D*_p_ and *thr* levels were determined by automatic and manual thresholding options, respectively.

## Results

### Genetic variability

All eight microsatellite loci tested within 13 populations have different levels of polymorphism in terms of number of alleles (ranging from moderately polymorphic, at five (Bcar181) to highly polymorphic at 16 (Bcar9)) and allele size range, as presented in Table 2. At locus Bcar73, allele 113 appeared to be fixed in male individuals of the SY5 strain, as reported by Isasawin et al. (2014). Linkage disequilibrium (LD) and Hardy-Weinberg equilibrium tests (HWE) were therefore performed for seven microsatellite loci for all populations. No significant evidence of LD among all loci was detected. After Bonferroni correction (Rice 1989), 28 out of 91 comparisons of loci and populations significantly deviated from the HWE results.

Overall genetic variations detected in each population is summarized in Table 3. *Bactrocera carambolae* samples collected from Southeast Asia showed relatively higher genetic variation than the introduced population (PR) for all parameters (i.e., *n*_a_, *n*_e_, *n*_r_, *A*_r_, *n*_p_, *A*_p_, *H*_O_ and *H*_E_). In addition, the values of genetic variation of three *Bactrocera dorsalis* populations, two unidentified populations, and the SY5 strain were in the same range as *Bactrocera carambolae*. The *n*_p_ values were observed in all populations, except for the NS, DK and PR populations, varying from 0.125 (DP, JK, KS and SY5) to 0.625 (RB) per locus. Likewise, rare alleles (allele frequency less than 0.05) were also detected in all populations (*n*_r_: ranging from 0.375 to 1.875 per locus), except KS. The average *H*_E_ values varied from 0.185 (PR) to 0.668 (RB). However, a deficiency in the average *H*_O_ values was found in all populations. Inbreeding (*F*_IS_) was detected in all populations, ranging from 0.095 to 0.628. The average for null alleles was 0.12, varying from low (0.01) to high frequency (0.25), which may contribute to the deficiency of heterozygosity observed in the given populations.

**Table 3.** Genetic variation among thirteen populations.

Sample	*n*_a_	*n*_e_	*n*_p_	*A*_p_	*n*_r_	*A*_r_	*H*_O_	*H*_E_	*F*_IS_
NS	3.375	1.980	0.000	0.000	0.625	0.032	0.202	0.410	0.532
PK	5.250	3.318	0.250	0.024	1.375	0.030	0.436	0.589	0.232
DP	5.000	3.257	0.125	0.059	0.625	0.030	0.381	0.648	0.386
JK	5.250	3.530	0.125	0.019	1.125	0.024	0.375	0.613	0.388
DK	4.625	2.937	0.000	0.000	0.875	0.024	0.347	0.528	0.258
NT	5.625	3.384	0.250	0.018	1.750	0.024	0.337	0.653	0.461
PR	2.000	1.324	0.000	0.000	0.375	0.015	0.152	0.185	0.095
BD	5.500	2.816	0.250	0.037	1.625	0.021	0.380	0.622	0.376
WK	5.750	3.257	0.375	0.092	1.875	0.026	0.406	0.660	0.393
RB	5.375	3.302	0.625	0.031	0.875	0.030	0.259	0.668	0.628
CM	4.250	2.466	0.250	0.074	0.750	0.037	0.387	0.572	0.315
KS	3.375	2.163	0.125	0.111	0.000	0.000	0.385	0.459	0.119
SY5*	3.286	1.952	0.125	0.016	1.000	0.032	0.317	0.433	0.274

*This data is calculated by using seven loci because locus Bcar73 is Y-pseudo linked.

*n*_a_, mean number of alleles; *n*_e_, mean effective number of alleles, 1/(1-*H*_E_); *n*_p_, mean number of private alleles; *A*_p_, mean frequency of private alleles; *n*_r_, mean number of rare alleles (allele frequency < 0.05); *A*_r_, mean frequency of rare alleles; *H*_O_, mean observed heterozygosity; *H*_E_, mean expected heterozygosity; *F*_IS_, mean inbreeding coefficient

### Population structure

Genetic differentiation among 13 populations was measured by the fixation index (*F*_ST_), as shown in Table 4. Among seven populations, the pairwise *F*_ST_ values range from 0.134 (between PK and JK) to 0.631 (between DK and PR). Genetic differentiation was relatively high between native and introduced populations, ranging from 0.444 to 0.631. The introduced population (PR) was most similar to population PK (*F*_ST_ = 0.444) and JK (*F*_ST_ = 0.448). Within native areas of *Bactrocera carambolae*, the pairwise *F*_ST_ values ranged from 0.134 (PK and JK) to 0.344 (PK and DK). On the other hand, the pairwise *F*_ST_ values among samples of *Bactrocera dorsalis* varied from 0.210 (RB and CM) to 0.357 (CM and KS). Without PR, the degree of genetic differentiation between *Bactrocera carambolae* and *Bactrocera dorsalis* ranged from 0.181 (DP and RB) to 0.524 (NS and KS). The SY5 strain was revealed to be genetically closest to JK (*F*_ST_ = 0.278). The degree of pairwise *F*_ST_ among pairs of the SY5 strain and others varied from 0.372 (SY5 and DP) to 0.507 (SY5 and PR) (Table 4).

**Table 4.** Significant pairwise *F*_ST_ among 13 populations.

Population	NS	PK	DP	JK	DK	NT	PR	BD	WK	RB	CM	KS	SY5
NS													
PK	0.296												
DP	0.274	0.162											
JK	0.217	0.134	0.169										
DK	0.288	0.344	0.287	0.300									
NT	0.329	0.248	0.188	0.241	0.206								
PR	0.596	0.444	0.495	0.448	0.631	0.564							
BD	0.334	0.240	0.228	0.176	0.368	0.264	0.491						
WK	0.345	0.216	0.163	0.197	0.336	0.273	0.486	0.194					
RB	0.404	0.260	0.181	0.234	0.339	0.204	0.582	0.202	0.162				
CM	0.395	0.321	0.254	0.290	0.401	0.249	0.636	0.259	0.200	0.210			
KS	0.524	0.402	0.322	0.347	0.368	0.168	0.738	0.352	0.317	0.256	0.357		
SY5	0.468	0.389	0.372	0.278	0.381	0.343	0.507	0.348	0.321	0.354	0.409	0.414	

**Figure 2.**
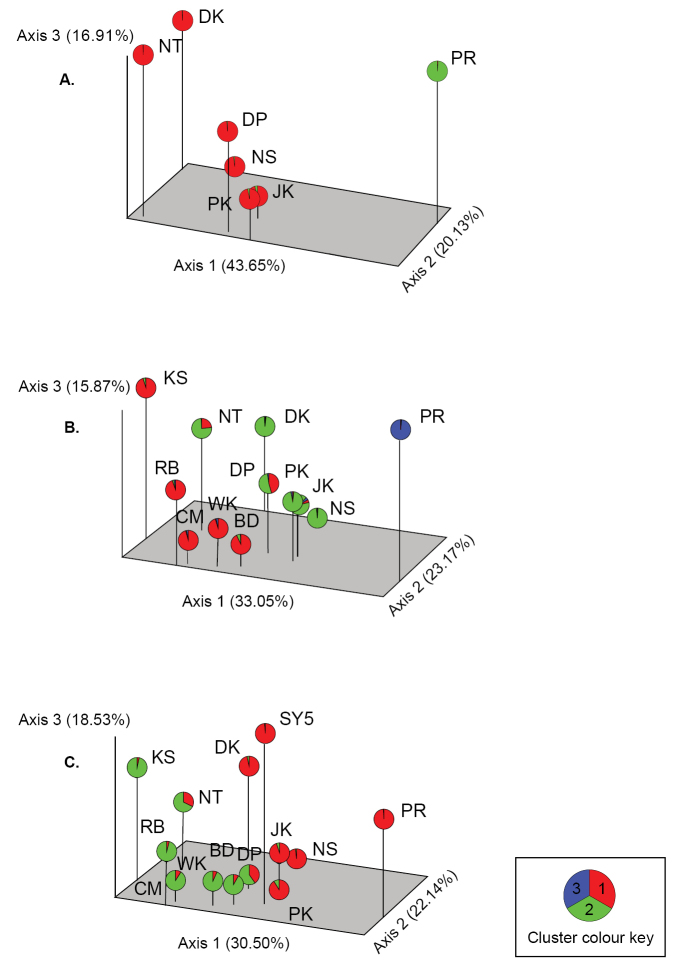
Three-dimensional plot of Principal Coordinate Analysis (PCoA) and STRUCTURE analysis. **A** the planes of the first three principal coordinates explain 43.65%, 20.13%, and 16.91% of total genetic variation, respectively, for seven *Bactrocera carambolae* populations using eight SSRs **B** the planes of the first three principal coordinates explain 33.05%, 23.17%, and 15.87%, respectively, for *Bactrocera carambolae* and *Bactrocera dorsalis* groups using eight SSRs **C** the planes of the first three principal coordinates explain 30.50%, 22.14%, and 18.53%, respectively, for the SY5 strain and wild populations using seven SSRs. Pie graphs, consisting of different colored sections, represent co-ancestor distribution of 185, 289, and 321 individuals in **A** two, **B** three, and **C** two hypothetical clusters, respectively.

STRUCTURE analysis demonstrated the proportion of co-ancestry (*Q*) distributed in hypothetical clusters (*K*) whereas PCoA illustrated the genetic divergence of fruit fly populations in multidimensional space, as shown in Figure 2. Among seven *Bactrocera carambolae* populations, the Delta *K* value (Evanno et al. 2005) was determined to be *K* equals two (*K* = 2) as the optimal number. At *K* = 2, genetic clusters were divided into two groups: native and introduced *Bactrocera carambolae*. Cluster 1 contained all native populations (NS (*Q* = 0.979), PK (*Q* = 0.969), DP (*Q* = 0.984), JK (*Q* = 0.953), DK (*Q* = 0.991), and NT (*Q* = 0.995)) whereas the introduce population (PR) was distinguished, forming its own cluster (*Q* = 0.988) (Figure 2A). This subdivision corresponded to the first axis (44% of total variation) of the principal coordinate.

When three additional populations of *Bactrocera dorsalis* (RB, CM and KS) and two unidentified populations (BD and WK) were included in the STRUCTURE analysis, the optimal number for *K* was three. Genetic clusters were separated into two groups: *Bactrocera dorsalis* belonged to cluster 1 while *Bactrocera carambolae* belonged to clusters 2 and 3 (Figure 2B). Six native populations of *Bactrocera carambolae* shared genetic memberships in cluster 2 (NS (*Q* = 0.976), PK (*Q* = 0.954), DP (*Q* = 0.519), JK (*Q* = 0.910), DK (*Q* = 0.970), and NT (*Q* = 0.758)). However, PR formed its own cluster, cluster 3 (*Q* = 0.986). Three *Bactrocera dorsalis* samples (RB (*Q* = 0.927), CM (*Q* = 0.955), and KS (*Q* = 0.952)) were grouped into the same genetic memberships (cluster 1) with two unidentified populations BD (*Q* = 0.927) and WK (*Q* = 0.953). Co-ancestor distribution between the *Bactrocera carambolae* and *Bactrocera dorsalis* clusters (*Q* ≥ 0.001) was observed in populations DP (*Q* = 0.457) and NT (*Q* = 0.236). Using PCoA, the first plane (33% of total variation) of multidimensional space also separated PR from the rest of the populations. Although the other two axes (23% and 16%, respectively) did not clearly divide samples into two groups in accordance with the STRUCTURE results, the group of *Bactrocera dorsalis* appeared to be plotted separately from the *Bactrocera carambolae* group.

**Figure 3.**
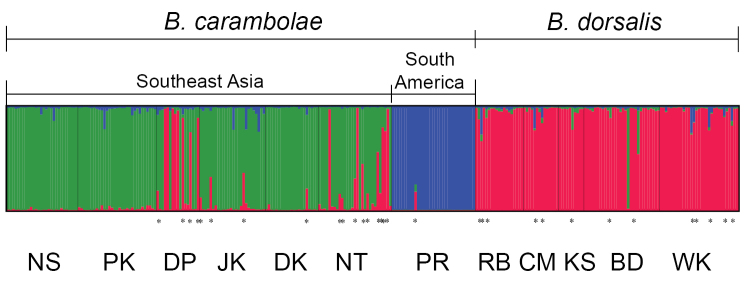
The individual admixture plot for *K* = 3. Each bar reveals a single individual. Each color of bars represents each genetic cluster. Samples of *Bactrocera carambolae* belong to clusters 2 and 3 (green and blue, respectively) while samples of *Bactrocera dorsalis* belong to cluster 1 (red). Potential hybrids have a proportion of genetic cluster (*Q*) between 0.100 to 0.900 (0.100 ≤ *Q* ≤ 0.900) as identified with asterisk (*).

The individual-admixture plot for *K* = 3 is presented in Figure 3. Individuals contained in the proportion of genetic cluster (*Q*) between 0.100 to 0.900 (0.100 ≤ *Q* ≤ 0.900) were identified as admixed individuals (or potential hybrids). Among the *Bactrocera carambolae* group, 18 of 185 individuals (9.73%) were admixed individuals. On the other hand, 13 of 104 individuals (12.5%) of the *Bactrocera dorsalis* group were admixed individuals. Pure individuals (*Q* > 0.900) were identified between the two groups. Eight individuals from the *Bactrocera carambolae* group were labelled as *Bactrocera dorsalis* whereas one individual from the *Bactrocera dorsalis* group was indicated to be *Bactrocera carambolae*.

Adding the SY5 strain to the genetic cluster analysis, two was the optimal number for *K.* At *K* = 2, genetic clusters formed two groups likely corresponding to *Bactrocera carambolae* and *Bactrocera dorsalis* groups. Six populations of *Bactrocera carambolae* (NS (*Q* = 0.981), PK (*Q* = 0.913), JK (*Q* = 0.948), DK (*Q* = 0.964), and PR (*Q* = 0.993) belonged to cluster 1 (Figure 2C). The SY5 strain was also a member of the *Bactrocera carambolae* cluster (*Q* = 0.988). Cluster 2 consisted of the *Bactrocera dorsalis* group (CM (*Q* = 0.909), RB (*Q* = 0.960), KS (*Q* = 0.964), BD (*Q* = 0.918), and WK (*Q* = 0.933)). However, populations DP (*Q* = 0.596) and NT (*Q* = 0.677) also shared membership in this cluster and became an admixture structure. The principal coordinates did not clearly delineate the two clusters using the three-dimensional plot. The PR population was still isolated from the others while the *Bactrocera dorsalis* group seemed to form a peripheral group.

Analysis of Molecular Variance (AMOVA) was used to study the hierarchical structure of populations for different scenarios (Table 5). Overall, up to 23% of variation was attributed to the differences among groups whereas approximately 52% to 62% of variation was attributable to differences within populations. In scenarios 1 and 2, populations of *Bactrocera carambolae* were divided into subpopulations according to micro- and macro- geographies. At the micro-geographical level, seven populations were divided into five groups: Sumatra, Indonesia (NS and PK); Java, Indonesia (DP and JK); Malaysia (DK); Thailand (NT); and Suriname (PR). Non- significant differences among those groups were detected (scenario 1: *P* = 0.1877). Likewise, when all seven populations were divided based on macro-geography (Southeast Asia vs. South America), a non-significant difference between groups was still observed (scenario 2: *P* = 0.1953). A test of genetic homogeneity between *Bactrocera carambolae* and *Bactrocera dorsalis* is presented in scenario 3, illustrating a significant difference (*P* = 0.0479). Even though BD and WK were included in the *Bactrocera dorsalis* group, a significant difference was still detected (scenario 4: *P* = 0.0156). In scenarios 5 to 7, the SY5 strain was included to compare the genetic variation among other samples. The results revealed a non-significant difference between *Bactrocera carambolae* and the SY5 strain (scenario 5: *P* = 0.2483) and among *Bactrocera carambolae*, *Bactrocera dorsalis* and the SY5 strain (scenario 6: *P* = 0.0694). However, a significant difference was indicated when BD and WK were included in the *Bactrocera dorsalis* group (scenario 7: *P* = 0.0342). The last two scenarios was set following the STRUCTURE results (Figures 2B and 2C, respectively). Significant differences were detected in both scenarios (*P* = 0.0010 and *P* = 0.0039, respectively).

**Table 5.** Analysis of molecular variance (AMOVA).

Scenario*	**Among groups**			**Among populations within groups**			**Within populations**		
	***V*_a_**	**Percentage**	***P***	***V*_b_**	**Percentage**	***P***	***V*_c_**	**Percentage**	***P***
1	0.2533	11.8	0.1877	0.5952	27.72	<0.0001	1.2990	60.49	<0.0001
2	0.5817	23.21	0.1953	0.6256	24.96	<0.0001	1.2990	51.83	<0.0001
3	0.1833	7.98	0.0479	0.7805	33.98	<0.0001	1.3335	58.04	<0.0001
4	0.1450	6.26	0.0156	0.7282	31.46	<0.0001	1.4416	62.28	<0.0001
5	0.1377	6.15	0.2483	0.8262	36.88	<0.0001	1.2767	56.98	<0.0001
6	0.1744	7.69	0.0694	0.7810	34.47	<0.0001	1.3105	57.84	<0.0001
7	0.1607	6.98	0.0342	0.7288	31.67	<0.0001	1.4121	61.35	<0.0001
8	0.3298	13.96	0.0010	0.5910	25.02	<0.0001	1.4416	61.02	<0.0001
9	0.139	6.08	0.0039	0.7498	32.57	<0.0001	1.4121	61.35	<0.0001

^*^Scenario 1: Sumatra, Indonesia (NS and PK); Java, Indonesia (DP and JK); Malaysia (DK); Thailand (NT); and Suriname (PR) Scenario 2: Southeast Asia (NS, PK, DP, JK, DK, and NT) and South America (PR) Scenario 3: *Bactrocera carambolae* (NS, PK, DP, JK, DK, and NT) and *Bactrocera dorsalis* (RB, CM, and KS) Scenario 4: *Bactrocera carambolae* (NS, PK, DP, JK, DK, and NT) and group of *Bactrocera dorsalis* (RB, CM, and KS), and BD and WK Scenario 5: *Bactrocera carambolae* (NS, PK, DP, JK, DK, NT, and PR) and the SY5 strain Scenario 6: *Bactrocera carambolae* (NS, PK, DP, JK, DK, NT, and PR) and *Bactrocera dorsalis* (RB, CM, and KS) and the SY5 strain Scenario 7: *Bactrocera carambolae* (NS, PK, DP, JK, DK, NT, and PR); group of *Bactrocera dorsalis* (RB, CM, and KS), BD, and WK; and the SY5 strain Scenario 8: STRUCTURE analysis (Figure 2B): cluster 1 (RB, CM, KS, BD, and WK), cluster 2 (NS, PK, DP, JK, DK, and NT), and cluster 3 (PR) Scenario 9: STRUCTURE analysis (Figure 2C): cluster 1 (NS, PK, JK, DK, PR, and SY5) and cluster 2 (RB, CM, KS, BD, WK, DP, and NT)

**Table 6.** Record of different host plants in Southeast Asia and Suriname for *Bactrocera carambolae* (edited from van Sauers-Muller 2005).

Hosts found in Southeast Asia only	Hosts found in Suriname only
*Annona montana* Macf.	*Anacardium occidentale* L.
*Annona muricata* L	*Spondias cytherea* Sonn.
*Thevetia peruviana* (Pers.) K. Schum	*Spondias mombin* L.
*Persea americana* Mill.	*Garcinia dulcis* (Roxb.) Kurz
*Artocarpus altilis* (communis) (Park.) Fosberg	*Malpighia punicifolia* L.
*Artocarpus heterophyllus* Lam.	Eugenia cf. patrisii Vahl
*Averrhoa bilimbi* L.	*Citrus sinensis* (L.) Osbeck
*Punica granatum* L.	
*Capsicum annuum* L.	
*Lycopersicon esculentum* Mill.	

### Isolation by distance (IBD)

The correlation between genetic and geographic distance was analyzed using only wild samples consisting of *Bactrocera carambolae* and *Bactrocera dorsalis* populations. The correlation between genetic and geographical distance became non-significant [*R*^2^ = 0.394, *P* = 0.106, *F*_ST_/(1-*F*_ST_) = 0.146 Ln (geographical distance) - 0.572] when *Bactrocera carambolae* samples were analysed. This fact indicates that there is no limitation of gene flow among *Bactrocera carambolae* across the region. Among *Bactrocera carambolae* and *Bactrocera dorsalis* populations, analysis showed significant correlation between genetic and geographical distances [*R*^2^ = 0.449, *P* = 0.002, *F*_ST_/(1-*F*_ST_) = 0.180 Ln (geographical distance) + (0.868)], even though only the PR sample was excluded (*R*^2^ = 0.119, *P* = 0.021).

### Genetic connectivity

**Figure 4.**
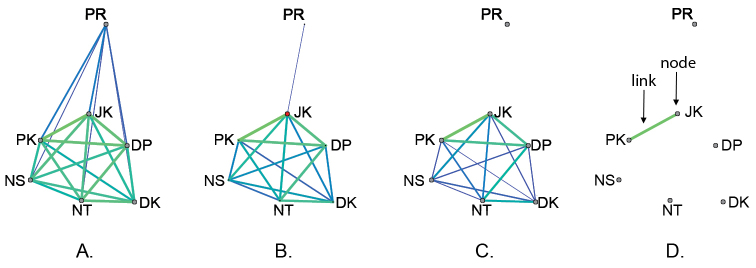
Simplified network of seven *Bactrocera carambolae* populations, and the sequential forms of cluster. The network was constructed using eight SSRs. Scanning was done for decreasing thresholds **A** is the fully connected network **B** is the percolation threshold (*D*_p_ = 0.52, with all links corresponding to distances superior to *D*_p_ excluded). JK plays an important role connecting between native and introduced populations **C**–**D** are the lower thresholds chosen (*thr* = 0.40 and 0.15, respectively) to reveal sub-structured network.

**Figure 5.**
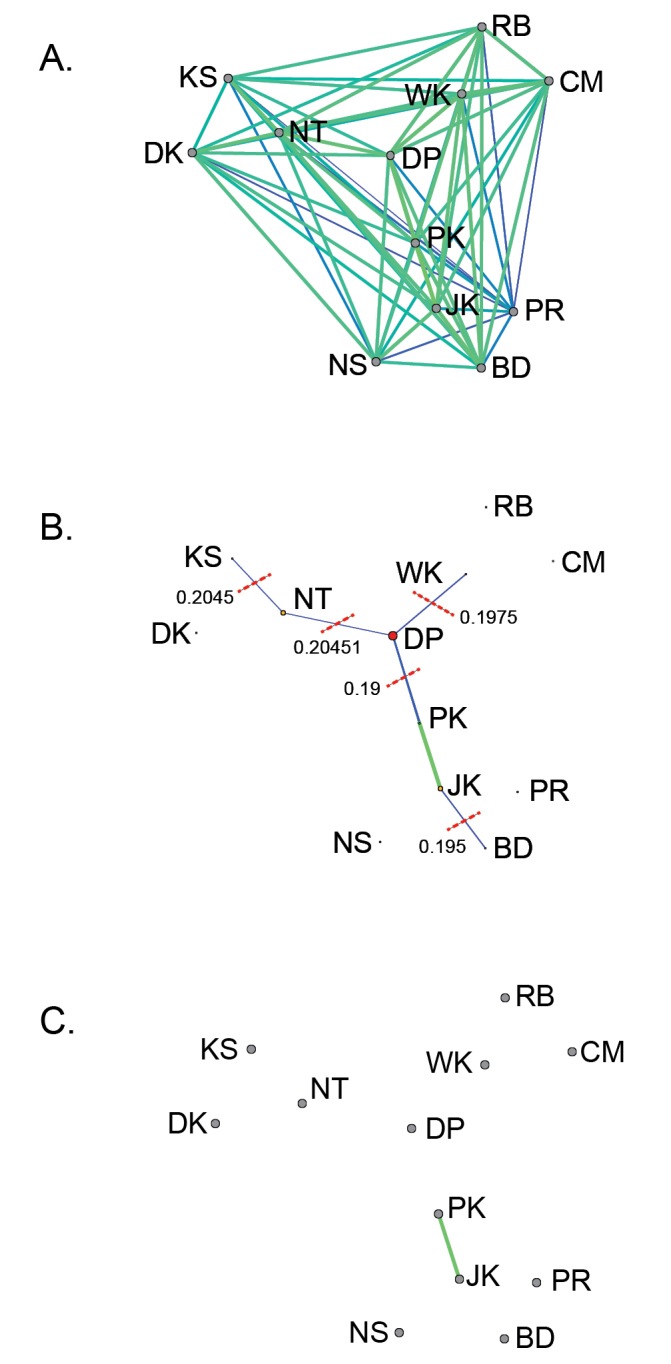
Simplified network of *Bactrocera carambolae* and *Bactrocera dorsalis* groups, and the sequential disconnection of the network. The network was constructed using eight SSRs. Scanning was done for decreasing thresholds **A** is the fully connected network **B** is the percolation threshold (*D*_p_ = 0.20, with all links corresponding to distances superior to *D*_p_ excluded). DP, JK, and NT are connecting between *Bactrocera carambolae* and *Bactrocera dorsalis* groups. Red dashed lines with number are corresponded to the threshold values, revealing serial disconnection of the network **C** is the lowest threshold (*thr* = 0.15).

**Figure 6.**
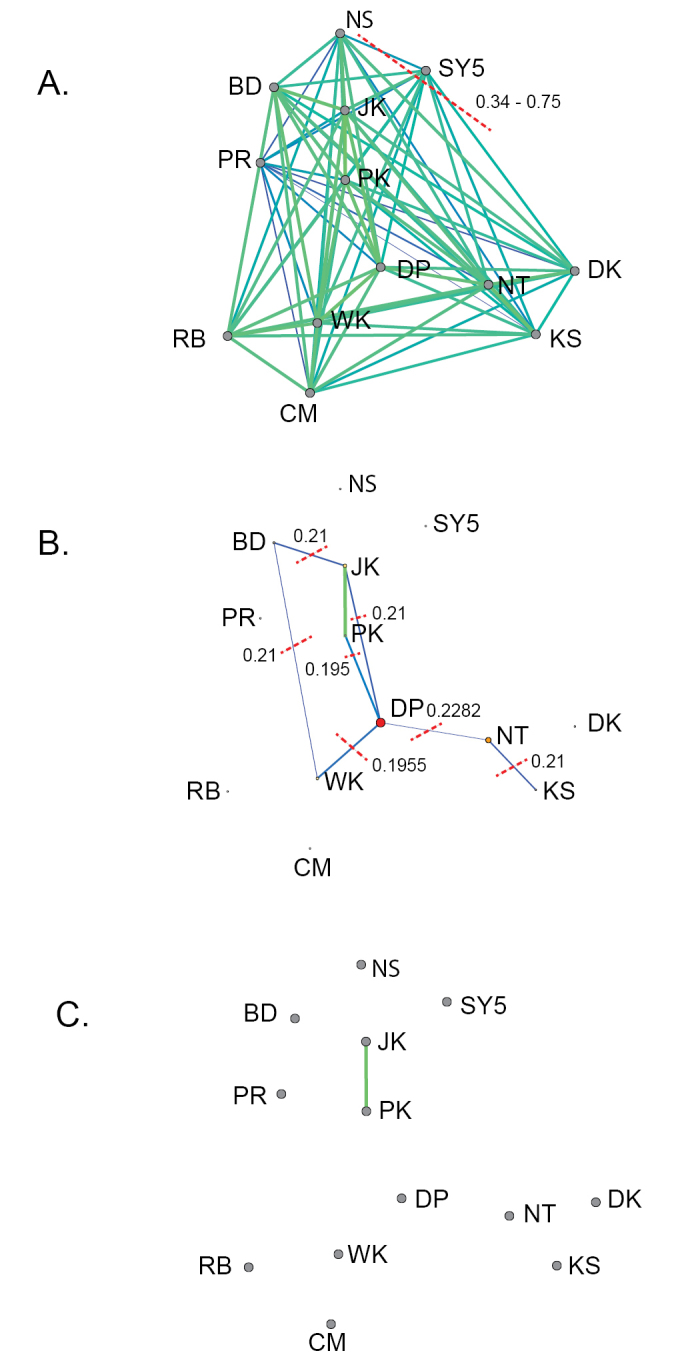
Simplified network of the SY5 strain and wild populations, and the sequential disconnection of the network. The network was constructed using seven SSRs. Scanning was done for decreasing thresholds **A** is the fully connected network **B** is the percolation threshold (*D*_p_ = 0.23, with all links corresponding to distances superior to *D*_p_ excluded). DP, JK, and NT are connecting between *Bactrocera carambolae* and *Bactrocera dorsalis* groups **C** is the lowest threshold (*thr* = 0.15). Red dashed lines with number are corresponded to the threshold values, revealing serial disconnection of the network.

Simplified networks were constructed for three different scenarios: (1) among seven *Bactrocera carambolae* populations, (2) among 12 populations belonging to *Bactrocera carambolae* and *Bactrocera dorsalis* clusters, and (3) among 13 populations, including the SY5 strain (Figures 4 to 6, respectively). Genetic distance metrics for the first and second data sets were estimated using eight microsatellite loci, but the third data set was analyzed using seven loci because the omitted locus is Y-pseudo linked in the SY5 strain (Isasawin et al. 2014). The networks were scanned for decreasing thresholds from the fully connected network to the percolation threshold (*D*_p_) and lower threshold chosen (*thr*) to reveal the sequential substructure at decreasing thresholds. The first scenario was constructed based on data among seven *Bactrocera carambolae* populations (Figure 4). The percolation threshold *D*_p_ = 0.52 showed the emergence of a connection between native and introduced populations (Figure 4B). The node of JK had a larger degree (Degree = 6.0) and betweenness-centrality (*BC* = 5.0) than others and played a role connecting between native and introduced populations (Suppl. material 1: Table 1). The scanning at decreasing thresholds illustrated sub-cluster of native and introduced populations (*thr* = 0.40; Figure 4C). Moreover, JK and PK populations were the first to be jointed in the network (*thr* = 0.15; Figure 4D).

For the second scenario, five more populations belonging to the *Bactrocera dorsalis* cluster were included in the network analyses (Figure 5). At the percolation threshold *D*_p _= 0.20, non-substructured system was observed (Figure 5B). Genetic connections between the *Bactrocera carambolae* and *Bactrocera dorsalis* groups were identified through the nodes of DP (Degree = 4; *BC* = 11.00), JK (Degree = 3; *BC* = 5.00) and NT (Degree = 2; *BC* = 5.00) (Suppl. material 2: Table 2). The network scanned below *D*_p_ demonstrated a serial disconnection of the system (Figure 5B). The first relationship of the network was detected between JK and PK populations (*thr* = 0.15; Figure 5C). To the contrary, the genetic links between native (JK and PK) and introduced (PR) populations of *Bactrocera carambolae* were revealed when increasing the threshold to *thr* = 0.55 (Degree = 11 and *BC* = 4.61) (Suppl. material 2: Table 2).

For the third scenario, the SY5 strain was added into the network to evaluate the genetic similarity between the SY5 strain and wild populations (Figure 6). The links between the SY5 strain and wild populations were detected above the percolation threshold (*thr* = 0.34 to 0.75) (Figure 6A). The SY5 strain shared greatest genetic similarity to JK (*thr* = 0.34). At the percolation threshold *D*_p_ = 0.23, non-substructured network was recognized (Figure 6B). Scanning below *D*_p_ showed disconnection of the system (Suppl. material 3: Table 3). Again, the first relationship of the network was detected between JK and PK populations (*thr* = 0.15; Figure 6C).

## Discussion

### Native vs introduced populations of *Bactrocera carambolae*

At the macro-geographical level, comparing among seven populations of *Bactrocera carambolae* from Southeast Asia and South America, the populations across the species’ native range possessed higher genetic variation than the introduced population, which is generally expected for invasive species. The first genetic connections between native and introduced populations were identified as Jakarta (JK) and Pekanbaru (PK), Indonesia. However, the genetic structure of the Suriname population (PR) (based on *F*_ST_, STRUCTURE, PCoA, and genetic network analysis) was differentiated from the Carambola fruit fly in Southeast Asia. These results were congruent with multilocus phylogenetic analysis established by Boykin et al. (2014). They deduced that factors and processes shaping the genetic variation and population structure of *Bactrocera carambolae* in the introduced area potentially include genetic drift during the colonization process and local adaptation. Based on the genetic data in this study, JK and PK are possible sources of the PR population. *Bactrocera carambolae* accidentally invaded South America, likely by tourists and trade from Indonesia to Suriname. Even though this species was detected in 1975, it took up to 21 years (1975–1996) to establish its new populations in other areas, expanding 500 km eastward and westward from the original point of introduction in Suriname (Malavasi et al. 2000, 2013). In the meantime, according to the report of van Sauers-Muller (2005), host plants of *Bactrocera carambolae* (e.g., guava, Malay apple, carambola, West Indian cherry, and mango) in Suriname were limited to backyards; agricultural production had not yet developed. Hosts for the fly were occasionally recorded to be different between native and introduced areas (van Sauers-Muller 2005), as shown in Table 6. Regarding that evidence, the conditions of habitats, including sufficient time for genetic drift to take effect, may be natural factors causing high genetic differentiation between native and introduced populations. Likewise, the same factors, including limitations of human activity, among four countries near Suriname may slow down species distribution in South America. This pattern is similar to the case of the related species *Bactrocera dorsalis* in several introduced areas such as Hawaii, Myanmar, and Bangladesh, in that their genetic variation and population structure were shaped by genetic drift and different local adaptations (Aketarawong et al. 2007). However, the current study included only one population from South America. Research using more samples, if available, from these regions should provide a better understanding of the demographic dynamics of the Carambola fruit fly within South America as well as between the two continents.

Within Southeast Asia, *Bactrocera carambolae* demonstrates high genetic variation and homogeneous population structure. West Java, in particular JK, is also a potential source of *Bactrocera carambolae* populations in Southeast Asia. JK showed relatively higher genetic variation and greater values of Degree and betweenness-centrality than other populations in the genetic network. Moreover, this area is important for the cultivation of star fruit and is a center for transportation to other cities and countries. The homogeneous genetic pattern of *Bactrocera carambolae* in native areas is similar to *Bactrocera dorsalis* collected from neighboring countries, including Thailand, Laos, and Cambodia (Aketarawong et al. 2007, 2014a, Schutze et al. 2012). Both not having limitations on human migration and the intensive cultivation of similar host plants could enhance gene flow, shaping genetic homogeneity among flies from nearby countries. Although the geography of Indonesia, Malaysia and Thailand is not entirely contiguous, increasing trade can promote the migration of insect pests within the country, as well as among other countries (Suputa et al. 2010). Therefore, effective quarantine measures are important to reduce the pest’s distribution in Southeast Asia.

Pairwise *F*_ST_ between native and introduced populations was significantly higher than zero. Approximately 13.4% to 32.9% and 44.4% to 63.1% of genetic diversity were results of genetic difference among populations within native areas and among populations between native and introduced areas, respectively. From the comparison of the genetic diversity of other closely related species using eight similar microsatellite loci with different primer sets as the current study, lower levels of genetic diversity (approximately 2% to 18%) were estimated from *Bactrocera dorsalis* (Aketarawong et al. 2014b). The most likely explanation of the situation involves a high level of gene flow and/or recent population divergence. In case of *Bactrocera carambolae*, it implies that the level of colonization of this invasive fly may not be as high as *Bactrocera dorsalis*. Alternatively, it can be deduced that populations, in particular between native and introduced areas, became diverted, congruent with studies of multilocus phylogeny using sequence analyses (Boykin et al. 2014). However, the *F*_ST_ value can be influenced by the geographical difference of sampling locations (Neigel 2002). Samples of *Bactrocera dorsalis* were collected at no more than 200 km intervals whereas in this study, *Bactrocera carambolae* populations were collected from locations varying from 20 (Depok) to 18,022 km (Paramaribo) from Jakarta, Indonesia. Research using more samples from different locations on a fine scale and/or more genetic markers may provide more details.

Departures from HWE were quite high and null alleles might be responsible for the departures. The average null allele frequency was moderate (0.12), varying from low (0.01) to high frequencies (0.25). The high departure from HWE was also observed in other study using a single set of microsatellite markers for different species such as the *Ceratitis* FAR complex (52.4%, Virgilio et al. 2013). We verified possible bias produced by the presence of null alleles in our data set. The STRUCTURE analysis was also repeated considering missing data as recessive homozygotes for the null alleles. We found that the effect of null alleles was negligible (Suppl. material 4: Figure 1).

### Species boundaries of *Bactrocera carambolae* and *Bactrocera dorsalis*

We found 44 alleles shared between *Bactrocera carambolae* and *Bactrocera dorsalis* while 14 and 27 alleles were detected in only *Bactrocera carambolae* and *Bactrocera dorsalis*, respectively. Coincidence of similar/different allele profiles between them at microsatellite loci may be due to several phenomena including retention of ancestral alleles in both sister species; substantial gene flow between the two species; size homoplasy (Estoup et al. 2002). For the latter case, homoplasy is possibly ruled out. It does not represent a significant problem for many types of population genetics (i.e., only 1–2% underestimation of genetic differentiation) considering only when microsatellite with high mutation rate and large population size together with strong allele size constraints were involved. The choice of more realistic mutation models than a common strict-Stepwise Mutation Model (SMM) should alleviate the homoplasy effect (review in Estoup et al. 2002, review in Selkoe and Toonen 2006).

Using the genetic cluster approach, assuming correlated allele frequencies in different clusters were likely to be similar due to migration or shared ancestral, species’ genetic structures were determined. We found admixed individuals (potential hybrids) in both clusters with relatively similar ratio (9.73% in *Bactrocera carambolae* cluster and 12.5% in *Bactrocera dorsalis* cluster). To avoid the shared descent, a stricter model using uncorrelated allele frequencies was tested. The results still presented species’ genetic cluster and admixed individuals (Suppl. material 4: Figure 1). Genetic connectivity also revealed that populations DP, JK, and NT in the *Bactrocera carambolae* cluster and population WK in the *Bactrocera dorsalis* cluster consisted of several admixed individuals, playing the role of linker between *Bactrocera carambolae* and *Bactrocera dorsalis* in the genetic network. Comparing to *Bactrocera dorsalis* and the junior synonym, *Bactrocera papayae*, they share better genetic profiles (Schutze et al. 2012, Krosch et al. 2013, Aketarawong et al. 2014b). Weak and no genetic structure were presented using both similar (but different primer sets) (Aketarawong et al. 2014b) and different microsatellite loci (Krosch et al. 2013). It was observed that a single cluster dominated the ancestral of all samples, when uncorrected allele frequencies were assumed in the analysis (Krosch et al. 2013). Using a different assumption of allowing similar allele frequencies between populations, the population’s genetic structure was observed rather than species and admixed individuals were found among population clusters (Aketarawong et al. 2014b).

The current study therefore provided additional evidence to support an incomplete reproductive isolation between *Bactrocera carambolae* and *Bactrocera dorsalis* (Wee and Tan 2005b, Augustinos et al. 2014, Isasawin et al. 2014). Boundaries between *Bactrocera carambolae* and *Bactrocera dorsalis* may be semipermeable, varied as a function of genome region. Alleles at microsatellite loci used in this study could be introgressed between two species rather than other nuclear and mitochondrial DNA sequences (Boykin et al. 2014). To achieve a clearer picture of species boundary, genome-wide comparisons (ranging from modest number of microsatellite loci to full genome sequences, transcriptome, or SNPs analysis) between recently diverged forms or species should be performed. This may not only offer patterns of differentiation across the genome, but also define the dynamics of species boundary (Harrison and Larson 2014).

### Implication of pest control using SIT for *Bactrocera carambolae*

Isasawin et al. (2014) reported a proof of concept to characterize and use the new genetic sexing Salaya5 strain (SY5) for controlling *Bactrocera carambolae*. At that time, two wild populations of *Bactrocera carambolae* were then included in the experiment. However, this study was focusing on how it would be possible to use the SY5 strain for pest control on a broader level, not only locally. Therefore, more samples of *Bactrocera carambolae* from native and introduced areas (i.e., Indonesia, Malaysia, Thailand, and Suriname) were included. More analyses on genetic variation, population structure and genetic network were performed between the SY5 strain and wild populations. We found that the SY5 strain had genetic variation, population structure, and genetic similarity comparable to *Bactrocera carambolae*, rather than *Bactrocera dorsalis*, in Southeast Asia. The results strongly confirmed that the Salaya5 strain had not diverted away from its original genetic makeup. Under laboratory condition, at least 12 generations apart, selection did not strongly affect genetic compatibility between the strain and wild populations. Therefore, the SY5 strain could be included in the pest control programs using male-only SIT for control *Bactrocera carambolae* at local and regional levels. However, an actual mating test is still required between the strain and samples from introduced populations.

SIT is a species-specific control method that can deliver environmental benefit. However, it may be restricted where at least two major target pests coexist and need to be controlled. Releasing sterile males of only one target may not ensure a reduction of all problems (Alphey et al. 2010). *Bactrocera carambolae* and *Bactrocera dorsalis* were reported to be sympatric species in some areas (e.g., Kalimantan). Although several lines of evidence suggested that both species showed mating compatibility to some degree (McInnis et al. 1999, Schutze et al. 2013, Isasawin et al. 2014), release of either sterile genetic sexing Salaya1 or Salaya5 strain may be not enough to control both target pests. The release both of the sterile genetic sexing Salaya5 and of Salaya1 strains for controlling *Bactrocera carambolae* and *Bactrocera dorsalis*, respectively, should maximize the success of SIT programs. A simulation of mating competitiveness tests and field operation, when releasing two species together, will be further required to gain knowledge for confirmation of the proposed idea.

In order to identify the fruit fly samples, although microsatellite data showed significantly different population structure of the two species, eight of 185 individuals (4.32%) and one of 104 individuals (0.96%) belonging to the *Bactrocera carambolae* and *Bactrocera dorsalis* clusters were identified as opposite to their original assumed identity. At the individual level, microsatellite data in this study may not provide definitive data for studying systematic questions of incipient species. However, at the population level, microsatellite data can be used to distinguish species. This is similar to the case of the *Ceratitis* FAR complex in that genetic clustering can solve three species’ statuses whereas other data (i.e., morphology, phylogenetics based on DNA sequence analyses, and niche) cannot (Virgilio et al. 2013). In this study, the two unidentified populations should be good examples to support this particular advantage of using microsatellite markers. Therefore, a combination of microsatellite data with other approaches should be better than a stand-alone approach to define and confirm individual and/or population.

## Conclusion

Pattern of connectivity and population structure, based on microsatellite DNA markers, showed that *Bactrocera carambolae* from an introduced population genetically differs from populations from the native range. Genetic drift during colonization process and local adaptation may be factors shaping its genetic diversity and population structure. However, only sampling from South America might not be sufficient to trace back the process of colonization within and between continents. West Sumatra (Pekanbaru-PK) and Java (Jakarta-JK) were identified as sources of the Suriname population, congruent with historical record of human migration between the two continents. A different pattern of population structure was observed in *Bactrocera carambolae* within native range, where free human movement and trading can promote genetic homogeneity. Between *Bactrocera carambolae* and *Bactrocera dorsalis* groups, potential hybrids provide evidence through individual-based admixture plots. This was additional supportive data suggesting that reproductive isolation between *Bactrocera carambolae* and *Bactrocera dorsalis* is somewhat leaky. Although morphological characterization and several nuclear and mitochondrial markers revealed distinct species, the hypothesis of semipermeable species boundaries between them cannot be rejected. Alleles at microsatellite loci could be introgressed rather than other nuclear and mitochondrial sequences. Regarding the final conclusion on pest management aspect, the genetic sexing Salaya5 strain for *Bactrocera carambolae* had not diverted away from its original genetic makeup (JK) and other neighbor populations. Under laboratory condition, at least 12 generations apart, selection did not strongly affect genetic compatibility between the strain and wild populations. Therefore, the Salaya5 strain could be possible to include in the pest control programs using male-only SIT in local and regional levels, although an actual mating test is still required between the strain and samples from introduced populations.

## Supplementary materials

Supplementary material 1 Component data at the five successive thresholds used to illustrate Figure 4 Data type: measurement Explanation note: Component data are used to illustrate the structure of the subset of *Bactrocera carambolae* populations. The Highest Betweenness-centrality is highlighted in blue. This dataset is made available under the Open Database License (http://opendatacommons.org/licenses/odbl/1.0/). The Open Database License (ODbL) is a license agreement intended to allow users to freely share, modify, and use this Dataset while maintaining this same freedom for others, provided that the original source and author(s) are credited.Nidchaya Aketarawong, Siriwan Isasawin, Punchapat Sojikul, Sujinda Thanaphum

Supplementary material 2 Component data at the four successive thresholds used to illustrate Figure 5 Data type: measurement Explanation note: Component data are used to illustrate the structure of the subset of *Bactrocera carambolae* and *Bactrocera dorsalis* populations. The highest Betweenness-centrality is highlighted in blue. This dataset is made available under the Open Database License (http://opendatacommons.org/licenses/odbl/1.0/). The Open Database License (ODbL) is a license agreement intended to allow users to freely share, modify, and use this Dataset while maintaining this same freedom for others, provided that the original source and author(s) are credited.Nidchaya Aketarawong, Siriwan Isasawin, Punchapat Sojikul, Sujinda Thanaphum

Supplementary material 3 Component data at the four successive thresholds used to illustrate Figure 6 Data type: measurement Explanation note: Component data are used to illustrate the structure of the subset of the Salaya5 strain and wild populations. The highest Betweenness-centrality is highlighted in blue. This dataset is made available under the Open Database License (http://opendatacommons.org/licenses/odbl/1.0/). The Open Database License (ODbL) is a license agreement intended to allow users to freely share, modify, and use this Dataset while maintaining this same freedom for others, provided that the original source and author(s) are credited.Nidchaya Aketarawong, Siriwan Isasawin, Punchapat Sojikul, Sujinda Thanaphum

Supplementary material 4 Comparisons among three different the individual admixture plots Data type: measurement Explanation note: Comparisons among the individual admixture plots of 289 individuals, for K = 3, considering correlated allele frequency, uncorrelated allele frequency, and missing data as recessive homozygotes for the null alleles, respectively. This dataset is made available under the Open Database License (http://opendatacommons.org/licenses/odbl/1.0/). The Open Database License (ODbL) is a license agreement intended to allow users to freely share, modify, and use this Dataset while maintaining this same freedom for others, provided that the original source and author(s) are credited.Nidchaya Aketarawong, Siriwan Isasawin, Punchapat Sojikul, Sujinda Thanaphum
