# Supplementary material for: Gene flow and genetic structure of Bactrocera carambolae (Diptera, Tephritidae) among geographical differences and sister species, B. dorsalis, inferred from microsatellite DNA data
Source: Zookeys. 2015 Nov 26;(540):239–72. doi: 10.3897/zookeys.540.10058 (PMC4714072; doi:10.3897/zookeys.540.10058)
Supplement: Supplementary material 4 — Comparisons among three different the individual admixture plots [file zookeys-540-239-s004.doc]

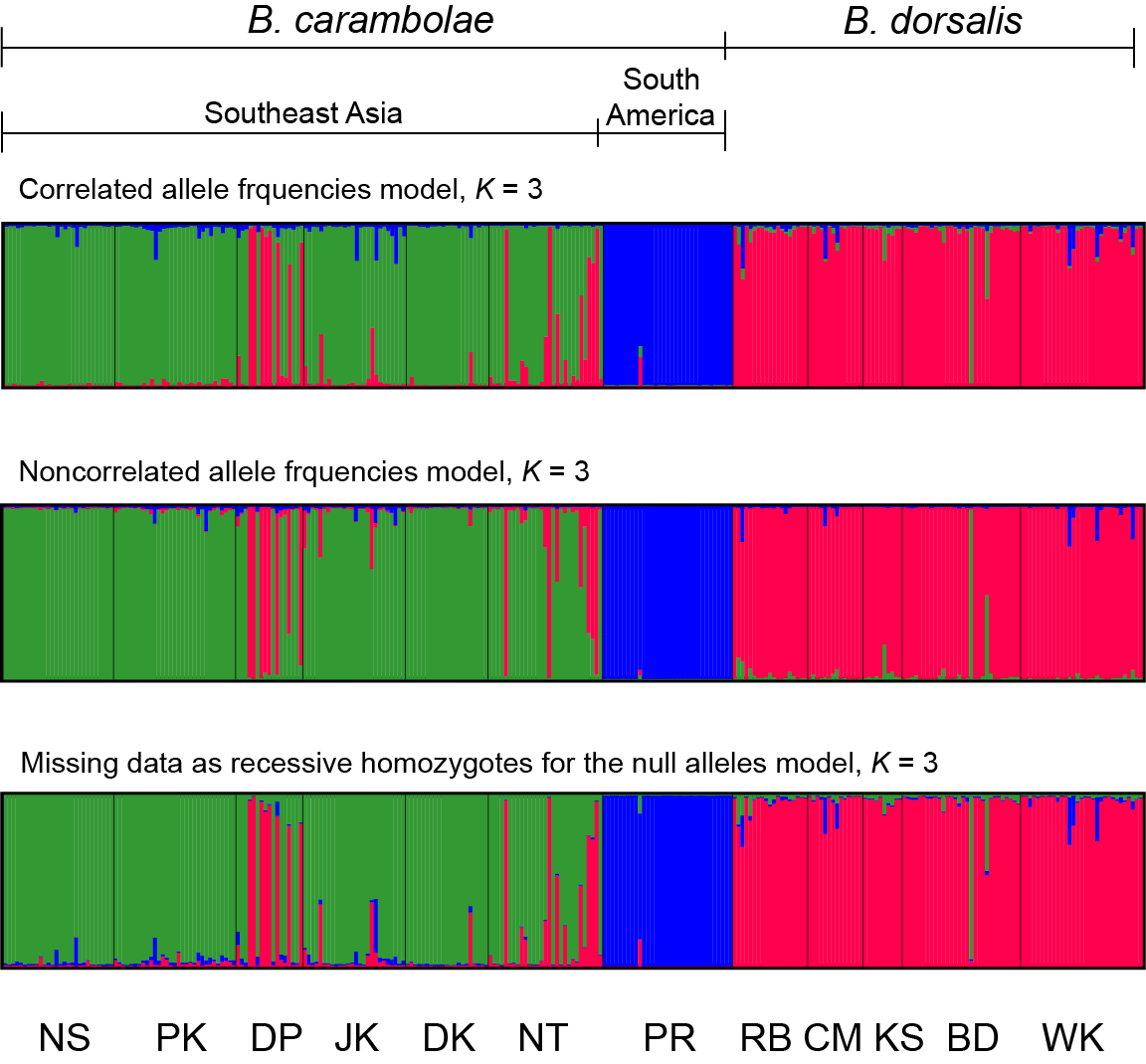


**Supplementary File 4: Figure 1** Comparisons among the individual admixture plots of 289 individuals, for *K* = 3, considering correlated allele frequency, uncorrelated allele frequency, and missing data as recessive homozygotes for the null alleles, respectively.
